# Disposable Soma Theory and the Evolution of Maternal Effects on Ageing

**DOI:** 10.1371/journal.pone.0145544

**Published:** 2016-01-11

**Authors:** Joost van den Heuvel, Sinead English, Tobias Uller

**Affiliations:** 1 Institute for Cell and Molecular Biosciences, Newcastle University, Newcastle Upon Tyne, NE4 5PL, United Kingdom; 2 Plant Sciences Group, Laboratory of Genetics, Wageningen University, Droevendaalsesteeg 1 6708PB, Wageningen, The Netherlands; 3 Edward Grey Institute, Department of Zoology, University of Oxford, Oxford, United Kingdom; 4 Behavioural Ecology Group, Department of Zoology, University of Cambridge, Downing Street, Cambridge CB2 3EJ, United Kingdom; 5 Department of Biology, Lund University, Lund, Sweden; CNRS, FRANCE

## Abstract

Maternal effects are ubiquitous in nature and affect a wide range of offspring phenotypes. Recent research suggests that maternal effects also contribute to ageing, but the theoretical basis for these observations is poorly understood. Here we develop a simple model to derive expectations for (i) if maternal effects on ageing evolve; (ii) the strength of maternal effects on ageing relative to direct environmental effects; and (iii) the predicted relationships between environmental quality, maternal age and offspring lifespan. Our model is based on the disposable soma theory of ageing, and the key assumption is thus that mothers trade off their own somatic maintenance against investment in offspring. This trade-off affects the biological age of offspring at birth in terms of accumulated damage, as indicated by biomarkers such as oxidative stress or telomere length. We find that the optimal allocation between investment in maternal somatic investment and investment in offspring results in old mothers and mothers with low resource availability producing offspring with reduced life span. Furthermore, the effects are interactive, such that the strongest maternal age effects on offspring lifespan are found under low resource availability. These findings are broadly consistent with results from laboratory studies investigating the onset and rate of ageing and field studies examining maternal effects on ageing in the wild.

## Introduction

Maternal effects describe a situation in which the phenotype of the mother has a causal effect on the phenotype of her offspring [1,2]. Many offspring characters are known to be subject to maternal effects, including physiology, morphology, behavior, and life history (reviewed in [3–7]). A familiar example is how maternal nutrition affects offspring size [8]. The most obvious reason for this is that females differ in the amount of resources they transfer to offspring, but maternal effects on offspring growth and development can also be driven by, for example, elevated cortisol in response to social stress, predation risk or population density (e.g., [9,10]). The phenotypic consequences of maternal effects often depend on offspring environment [11]. For example, offspring may mitigate negative effects of poor maternal nutrition in resource-rich environments (e.g., [12]) or exhibit adaptive plasticity in response to maternal cues [9,10], and might be expressed in a sex-specifically [9,13]. Thus, the expression of a maternal effect in terms of its contribution to variance in offspring phenotype is a function of both the maternal and offspring environment (e.g. [14–16]).

Maternal effects are often strongest early in life (e.g. [17]), but there is increasing evidence that they also have long-term consequences [4,18–20]. One intriguing observation is that a mother's physiological state can influence their offspring's lifespan and cause an increased risk of disease and poor health in late age, i.e., a higher rate of ageing [21–24]. Similar negative effects on health and reproduction have been observed in offspring from old mothers in both human and non-human animals (e.g. [25–27]). For example, daughters of old mothers show increased reproductive ageing compared to those of young mothers in wild great tits [25], and have lower lifetime reproductive success in wild house sparrows [28]. These maternal effects on offspring ageing may depend on the direct environmental conditions experienced by offspring.

The idea that ageing results from a trade-off between reproduction and survival is the basis of the evolutionary theories ageing such as the antagonistic pleiotropy [29] and disposable soma theory [30]. In the latter, ageing results in a physiological trade-off between investment in somatic maintenance and investment in other biological functions [30]. Damage to cells and tissues arise both due to exposure to external challenges and because biological processes directly cause damage as a side-effect. For example, growth and differentiation results in release of free radicals that can damage DNA and other molecules and result in loss of cellular function [31–33]. Further theory was developed to include the physiological trade-off between early life growth and somatic maintenance [34,35] and the effects of these trade-offs in very early development, with late life effects [36] which holds in experimental cases [37]. Consequently, the damage that causes ageing under the idea of the disposable soma theory should begin already during embryonic development, which is supported by studies showing that maternal stress hormones deposited in the egg increase oxidative damage and reduce telomere length [38]. Importantly, at this stage in life, offspring rely on maternal nutrition to prevent and repair damage caused by developmental stress. We therefore propose that variation in maternal investment can increase or decrease somatic maintenance in offspring and thereby can generate maternal effects on offspring biological age at birth (or more generally up until the end of maternal resource investment). Indeed, recent studies of biomarkers of ageing suggest that ageing begins already in embryonic development, such that the biological age at birth is not equal for all offspring [39–42] and that early-life estimates of oxidative damage can predict lifespan [43–46]. Such investment may depend both on the mother's own age and her access to resources, which therefore may contribute to variation in ageing.

To address this possibility, we developed a simple model based on the disposable soma theory of ageing (e.g. [47]). Therefore, we assume that mothers face a trade-off between investment in their own somatic maintenance and investment in offspring somatic state (as defined by their biological age, or extent of damage, at birth). Although in this sense the maternal effect on offspring ageing is a direct function of reproductive investment, it is not obvious how this should vary with maternal state, such as nutrition levels, nor how important maternal effects will be relative to direct environmental effects. In an environment where resource availability is variable, mothers may adjust their reproductive allocation accordingly, such that offspring ageing depends on the maternal environment in addition to the environment offspring experience during their own lifespan. The presence of both maternal and direct environmental effects on damage makes it difficult to predict *a priori* the relationship between resource availability and investment in own versus offspring maintenance. Furthermore, if mothers adjust their reproductive allocation across their lifespan, the effects on offspring ageing may also vary with maternal age. Thus, our simple model could potentially capture a range of biologically relevant maternal effects on ageing that are increasingly supported by longitudinal and experimental data on humans and other animals (e.g.[25,28,42,48–52]).

## The Model

In this model, we consider how maternal effects on offspring survival and lifespan evolve in response to environmental heterogeneity and how these depend on variation in resource availability and the age of the mother. We use a simple life history model in which mothers face a trade-off between maternal maintenance and reproduction, with more allocation to reproduction resulting in offspring with lower biological age. This concept of biological age provide a composite measure of an individual's general state of health, as characterized by factors such as molecular damage to DNA, mitochondrial status and immunological status [53,54,55]. First, we describe the physiology of the individuals characterized by our model and the assumptions on which it is based. We then outline how we calculate optimal decisions– investment in maintenance versus reproduction (which defines an offspring's biological age at birth)– using backward iteration, and how these decisions depend on the environment and physiological state of individuals. This step can be considered to describe how maternal effects on ageing (i.e. offspring biological age at birth) might evolve. Lastly, we describe a set of theoretical experiments performed to study how these evolved decisions perform under different conditions (forward simulations). This allows us to address how the environmental state of mothers and offspring can influence the strength of any such maternal effects on ageing.

### Physiology of the organism

We characterize the organism by two state variables, developmental stage (*X*, juvenile or adult) and biological age (*D*, note that this is different from chronological age as explained above). Depending on its biological age (e.g., represented by levels of cellular damage that cause increased risk of mortality), an adult makes strategic decisions on how much resource to allocate to its own maintenance or reproduction, which is optimized using a dynamic program algorithm [56,57]. Optimization models are based on the assumption that natural selection will shape life histories in such a way that organisms evolve the allocation strategy that maximizes lifetime fitness across the environments that are encountered.

We consider an organism that undergoes development for a fixed number of time steps (*S*_*DEV*_), after which it matures and can start to reproduce. Throughout life, biological age increases with time as a function of resource allocation to somatic maintenance. Low investment in maintenance results in rapid increase in biological age. During the juvenile stage (*S<S*_*DEV*_), we assume that all available resources are allocated to maintenance and none to reproduction. Following maturity, resources can be allocated either to maintenance or reproduction. For simplicity, we do not explicitly consider allocation to growth during the juvenile period (instead we assume this is contained within the maintenance parameter), nor do we consider allocation to growth of reproductive tissue either before or after maturation. We return to these assumptions and how they can be relaxed in the discussion. Similar to a previous model [58], we model the increase of biological age as,

$$D_{t+1}=D_{t}+\Delta D_{t}\left(U\right)$$ (1a)

$$\Delta D_{t}\left(U\right)=\kappa \frac{\text{exp}\left(\omega U\right)}{\beta +\text{exp}\left(\omega U\right)}$$ (1b)

where Δ*D*_*t*_ denotes the increase in biological age (*D*) at time *t*, *κ* is the maximal increase of biological age per time step, ω the efficiency of somatic maintenance, *β* the half-saturation value for maintenance, and *U* the total amount of resource allocated to maintenance. When *β* (half-saturation value for maintenance) equals *exp(ωU)*, damage increase is equal to half of the maximal damage increase. More allocation to maintenance leads to less damage, while less allocation to maintenance increases damage levels (hence ω is negative, making the term *exp(ωU)* smaller with more allocation). Therefore, variation in allocation between individuals with similar chronological age results in increasingly different biological ages. Resource allocation to maintenance (*U*) or reproduction (*R*) depends both on the strategic choice of the organism (in terms of proportion of resources allocated to maintenance, *q*) and resource availability (*E*) as follows,

U(q,E)=qE (2a)

R(q,E)=(1−q)E (2b)

where the absolute amount of acquired resources, *E*, can vary between patches in the environment. Note that during the juvenile stage all resources are allocated to maintenance, and therefore *q* is set at 1.

Since our focus is on maternal effects on offspring ageing, we assume that allocation to reproduction has a direct effect on the biological age of offspring at birth. This is consistent with a growing literature showing that the early environment may contribute to ageing as measured by oxidative damage or telomere shortening [39,44,46]. We modeled the biological age at birth in a similar way as in Eq 1b, by replacing *U* by *R*. Therefore, the biological age of an offspring at birth, *D*_*0*_, from a mother that invests *R* amount of resource in reproduction is,

$$D_{0}(R)=\kappa \frac{\text{exp}(\omega R)}{\beta +\text{exp}(\omega R)}$$ (3)

This means that there is a trade-off between maternal maintenance and the quality of her offspring, where the latter is expressed in terms of biological age. Note that our period of maternal reproductive investment applies to a range of life-histories including egg production and fetal growth. All else being equal, mothers that invest more resources in reproduction will have offspring with a lower biological age at birth than mothers that invest fewer resources. Here, we assume the increase in biological age between offspring and adults is similar for a given amount of resources allocated to maintenance (i.e., parameters *κ*, ω and *β* are equal between Eqs 1b and 3, see also Fig G. Fig H and Fig I in S1 File). We also assume that an offspring is born every time step, i.e., there is baseline level of reproductive investment that cannot be foregone. In this model we do not allow females to adjust offspring number and hence any changes in reproductive investment directly affect only the quality of the offspring in terms of its biological age at birth. Our model is thus constructed such that we could focus on the trade-off between maternal and offspring health. Later extensions of the model, allowing for the number of offspring to be varied, could incorporate a quantity-quality trade-off in offspring as well.

We model evolution of investment in maintenance and reproduction in a variable environment, such that the environment of the mother may change with a fixed probability at every time step. In all the examples in this paper, there are five different types of environment (which could be considered patches of different quality connected by movement). Each environment has a different amount of resource present and the encounter probability of each type of environment is the same, i.e. 0.2. In the main model, the amount of resources from these patches are set to 0.1, 0.3, 0.5, 0.7 and 0.9 for the five different patches (*p*_*1*_ to *p*_*5*_, see Fig F in S1 File for demonstration that our conclusions are robust when we consider all values ranging from 0.3 to 1.2 at intervals of 0.1). Generally speaking, variation in resource availability and extrinsic mortality could affect age-dependent investment in maintenance versus reproduction. Under this model approach, the amount of investment in reproduction determines the level of damage in offspring and hence their biological age at birth. Patterns of ageing should therefore depend on both the current resource availability of the focal individual, and the resource availability and age of its mother. Exactly what these relationships will look like is not clear *a priori*, however. First, allocation is not necessarily positively correlated with acquisition under optimal strategies [59–61]. Second, the optimal allocation in a given environment may also depend on the expected future returns on investment, which is a function of biological age [62]. Third, because maternal effects generate variable and environment-specific initial states, these could have both immediate and long-term effects on optimal allocation strategies across ontogeny (e.g., [63]). Our model makes it possible to test the strength and direction of these effects for optimal investment strategies.

The mortality rate of an individual per time step, *M*, has a basal mortality parameter (*μ*_*b*_) and a biological-age-dependent mortality parameter (*μ*_*d*_), calculated following [64] as

$$M(D)=\mu_{b}\text{exp}(\mu_{d}).$$ (4)

### Calculation of optimal strategies by backward iteration

We use stochastic dynamic programming [57,65] to calculate optimal strategies based on individual state. In general, this model approach requires backward iteration from a time horizon to establish the optimal decisions for particular state variables which maximize future expected fitness, and subsequently generate predictions for particular scenarios using forward simulations. Thus, we first calculate, for each time step, the optimal decision of an adult (*q*, the proportion of acquired resource allocated to maintenance versus reproduction). The optimal value of *q* maximize fitness, (*F*_*J*_*(s*,*d*,*p*,*t)* for juveniles and *F*_*A*_*(d*,*p*,*t)* for adults), which depends on the developmental time (*s*), biological age (*d*), patch quality (*p*) and time (*t*). Note that the term for adult fitness does not contain *s*, since adults have completed their development.

We calculate fitness of a juvenile with development time (*s<*S_DEV_), biological age *d*, in patch *p*, at time *t* by

$$F_{J}\left(s,d,p,t\right)=\text{exp}\left(-M\right)\;\sum_{p^{\prime }=p_{i}}^{p_{s}}\;0.2F_{J}\left(s^{\prime },d^{\prime },p^{\prime },t+1\right),$$ (5)

where *p*’ indicate the patch in the subsequent time step, *s*’ the developmental time in the subsequent time step, *d’* the biological age in the subsequent time step and *t* the time since conception. Survival is equal to [*exp(-M)*] and the fitness measure was summed over the five types of environment, with the probability of 0.2 for encountering each environment (*p’*). When a juvenile reaches maturity (s = S_DEV_), it develops into an adult and at this point its fitness is equal to that of an adult who has yet to reproduce, i.e.:

$$F_{J}\left(S_{DEV},d,p,t\right)=\text{exp}\left(-M\right)\;\sum_{p^{\prime }=p_{i}}^{p_{s}}\;0.2F_{A}\left(d^{\prime },p^{\prime },t+1\right)$$ (6)

The fitness of an adult beyond maturity (s>S_DEV_) is calculated as a combination of an individual's current reproductive output and its expected future fitness if it survives to the next time step, given its updated state,

$$F_{A}(d,p,t)={\text{max}}_{q}\{\;\text{exp}(-M)\;\sum_{p^{\prime }=p_{i}}^{p_{s}}\;0.2\left[F_{J}(0,D_{0},p^{\prime },t+1)+F_{A}(d^{\prime },p^{\prime },t+1)\right]\}$$ (7)

where *F*_*J*_*(0*,*D*_*0*_, *p’*,*t+1)* refers to the biological age *D*_*0*_ of a newborn offspring, as calculated by Eq 3. The fitness is calculated over all considered values of the allocation parameter *q* (which in the current settings varies discretely between 0 and 1, with step size of 0.05). The results were qualitatively similar when we considered finer steps of *q* (0.01 or 0.001).

Our measure of fitness, also termed expected maximum accumulated reproductive success [34,35,58], was a combined measure incorporating the quality of offspring produced as well as expected future reproductive success based on changes in state due to the outcome of the current optimal decision. Since the quality of the offspring differed as a result of investment decisions, we accounted for offspring quality in our fitness measure by calculating the fitness of the offspring (following [58,59,61]; see SI for further information). The modelled trade off led to a fitness function with one optimum (see Supporting Information 1). Our measure of fitness was similar to the number of offspring required to maintain a population at equilibrium (*R*_*0*_). Using a measure of fitness that maximizes population growth rate (*r*) does not lead to qualitatively different results, but increases the relative investment into reproduction (see Supporting Information 2 for comparison with genetic simulation).

Eqs [5], [6] and [7] were solved by a dynamic programming algorithm using the parameters settings listed in Table 1. The fitness values at the time horizon (i.e. *F*_*J*_*(s*,*d*,*p*,*T*) and *F*_*A*_*(d*,*p*,*T*) where *T* is the time horizon) were taken to be 1. Iterating backwards in time, the fitness values and optimal allocation decisions converged on a single value [58]. Typically in dynamic optimization models, the maximum value of a state variable is never reached (in this case 1999 for biological age (D) when forward simulation is performed) and the steps in state space are always smaller than the steps caused by the decisions and environments. Therefore, both increasing the number of steps and taking a higher maximum state should not influence the outcome of the model qualitatively (see also [34]).

**Table 1:** Overview of parameters and variables with their respective numeric values.

Parameters	Description	Value(s)
S_DEV_	Chronological age at maturity	19
κ	Maximum increase of biological age	1000
ω	Maintenance efficiency	-4
β	Half-saturation value for maintenance	10
μ_b_	Basal mortality	0.005
μ_d_	Biological age dependent mortality	0.005
States		
S	Development time	0–20
D	Biological age	0–1999
E	Resource acquisition value ^a^	0.1, 0.3, 0.5, 0.7, 0.9
Other variables		
U	Resource allocated to maintenance	0–0.9
R	Resource allocated to reproduction	0–0.9
D_0_	Biological age at birth	0–1000
M	Mortality per time step ^b^	0 –∞
Q	Strategic value of allocation to maintenance or reproduction	0–1

^a^ acquisition takes five discrete values in the current model, but this can be adjusted based on user preferences

^b^ although theoretically biological age is unlimited in practice it is limited in this model by 1999 (see Eq 4).

## Forward Simulation

In the backward iteration described above, we calculated the optimal decisions, of relative allocation to maintenance (*q*) versus reproduction, for adults in different patches and in different states (i.e. different biological ages). We assumed that within the physiological constraints (as posed by Eqs [1–4] above), natural selection would have optimized these decisions. Hence, quantitative genetic parameters such as mutation rate, selection intensity, fitness peaks and the constraints posed by these phenomena are not considered (see [57] and references within for further discussion). These optimal decisions, which depend on the biological age of the mother and the maternal environment, were used in four simulated scenarios. We used these simulations to exemplify our points of interest, namely (i) the relationships between maternal resource availability or maternal age and offspring ageing (scenarios 1 and 2 below) and (ii) the strength of maternal resource or age effects on ageing relative to offspring direct environmental effects (scenarios 3 and 4 below).

**Fig 1:**
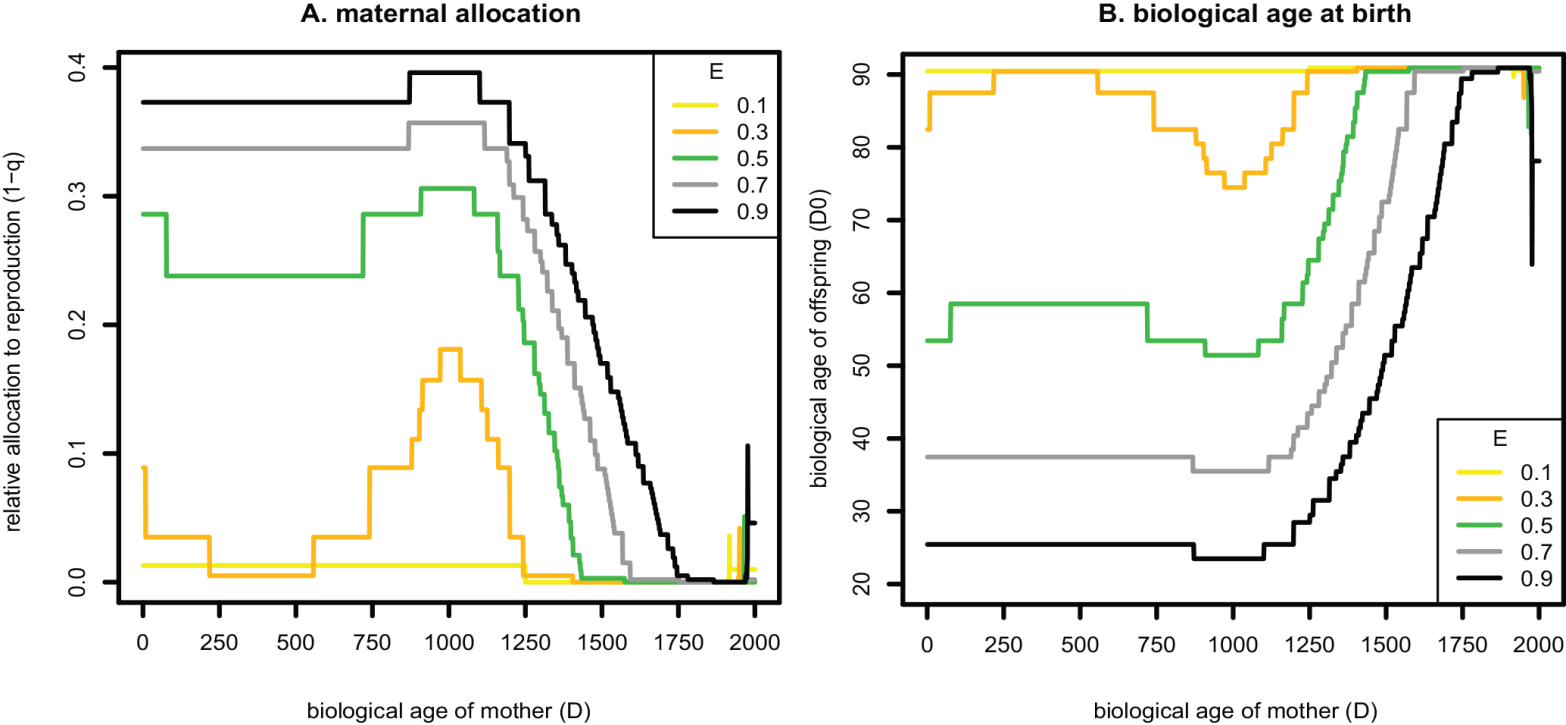
Optimal allocation strategies of mothers depending on their current nutrition and biological age. (A) Proportion of acquired resources allocated to offspring as a function of the resource environment and biological age of the mother. (B) As a consequence of the pattern in panel A, the resource environment availability and biological age of the mother affects the biological age of offspring at birth. Results are shown for five different levels of maternal resource environment (E) which are indicated by lines of different colours (see text for model details).

First, we simulated the effect of maternal environment, i.e., her resource acquisition, on the survival of the offspring under the evolutionary background condition used to derive the optimal strategy (i.e., equal probability of offspring encountering any of five different resource availabilities at any given time step). For this simulation, we kept maternal age constant such that all mothers have a biological age of 1200, which is representative of the allocation pattern for young mothers (with our model parameters, allocation is stable from age zero up to about a biological age of 1200; see Fig 1A). Second, we simulated the life courses of offspring in the evolutionary background condition, but with mothers of different biological age (five levels, D = 0, 400, 800, 1200, 1600). In this simulation, we kept resource acquisition constant so that all offspring come from mothers in the high resource environment (E = 0.9). Third, we simulated classes of offspring born from young mothers from five different types of resource environments (E = 0.1, 0.3, 0.5, 0.7, 0.9), but where the offspring encountered a constant resource environment at one of the five possible levels, i.e., a total of 25 classes of offspring. Fourth, we simulated classes of offspring born from mothers from five different ages, and where the offspring encountered one of five constant resource environments again, i.e., a total of 25 classes of offspring (with levels of biological age and resource availability described above). The last two simulations represented situations common in experimental studies, where performance is typically assessed in a single environment, and enabled us to investigate the relative effect sizes of maternal versus direct environmental effects and of maternal age, respectively.

For every above-mentioned group of offspring (i.e. combination of maternal age and offspring resource level, or of maternal resource levels and offspring resource level), 250,000 individual offspring were simulated. Parameter values were varied as described above (such as E during backwards iterations and E and D during forward simulations) or, if fixed, as listed in Table 1.

### Statistical analysis

To analyze the survival curves of the offspring from the four simulated scenarios, we fitted a Cox proportional hazard model [66] with the explanatory variables maternal resource environment, maternal age, and offspring environment (the latter only in simulations 3 and 4 where the environment is constant throughout life). Chronological age is defined as the number of time steps since maturity and is represented on the x-axis of the figures. From these models, we measured the proportion of total variation in offspring lifespan explained by the variable of interest (i.e. maternal and offspring environment and maternal age). Furthermore, we conducted a power analysis by bootstrapping the simulated individuals. We sampled 200 individuals from each group of offspring, after which a Cox proportional hazard test was fitted. This was done 500 times, and we assessed the proportion of instances in which a treatment group had a significantly different survival (α = 0.05) compared to a reference treatment (see results). We used this power analysis to exemplify how often, in a study designed to test for maternal effects on ageing, there was a significant difference in survival between treatment groups (when sampling 200 individuals). These results should only serve as illustration and not be taken to represent predictions for specific experiments.

## Results

Mothers consistently allocated more resources to reproduction versus somatic maintenance when they encountered high-resource environments compared to when they encountered low-resource environments (Fig 1A). Older mothers invested less in offspring compared to younger mothers. However, this was only apparent in higher resource environments (Fig 1A), resulting in an interaction between maternal resource environment and maternal age on allocation to offspring versus maternal maintenance. Therefore, biological age at birth (a measure of the amount of damage) was higher for offspring born from young mothers in low resource environments than for offspring born from young mothers in high resource environments, but this difference became gradually smaller for older mothers (Fig 1B).

**Fig 2:**
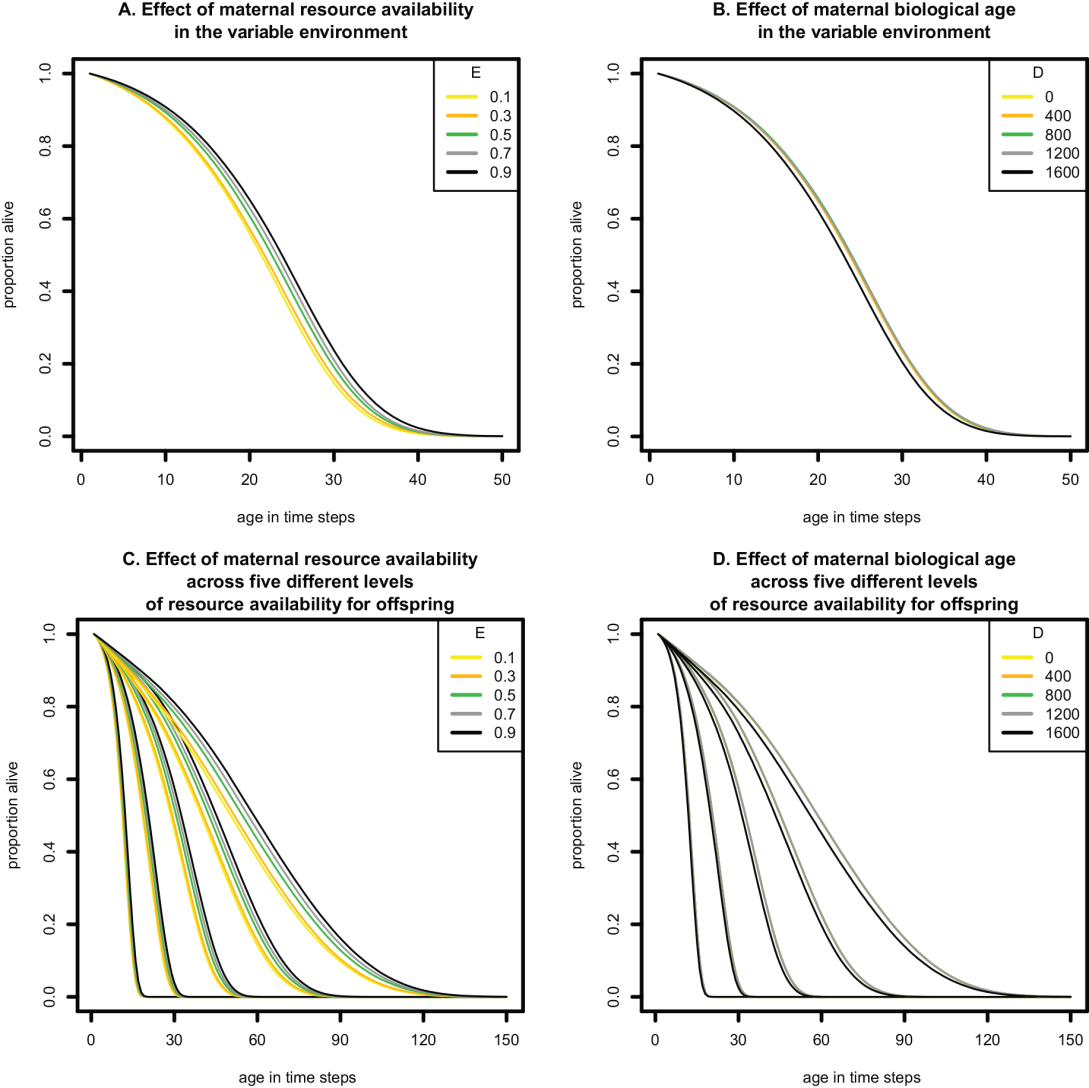
Survival curves of offspring in four simulated scenarios. The x-axes represent age in time steps during the forward simulation. (A) Survival of offspring born from middle age mothers (D = 1200) in patches of different resource levels (lines of different colours represent different values for maternal resource [E]) under the variable environment, i.e., when offspring experience unpredictable variation in resources throughout life, which is similar to the evolutionary background condition. (B) Survival of offspring born from mothers of different biological age (lines of different colours represent different values for maternal biological age [D]) in a high quality environment (E = 0.9) under variable environmental conditions, which is similar to the evolutionary background condition. (as in panel A). (C) Survival of offspring born from middle-age mothers (D = 1200) from different resource environments (lines of different colours represent different values for maternal resource [E]). Offspring encountering a single resource environment throughout life are grouped together, which results in five groups of survival curves from left to right (offspring experienced one of five patch types constantly through the rest of development, and the survival curves are therefore clustered into five distinct groups), each with the maternal resource availability indicated in colour (from low to high, i.e., E = 0.1, 0.3, 0.5, 0.7 and 0.9). (D) Survival of offspring born from mothers of different biological age (lines of different colours represent different values for maternal biological age [D]) in a high quality environment. Offspring encountering a single resource environment throughout life are grouped together, which results in five groups of survival curves from left to right (offspring experienced one of five patch types constantly through the rest of development, and the survival curves are therefore clustered into five distinct groups), each with the maternal age indicated in colour (from young to old, i.e., D = 0, 400, 800, 1200, 1600).

The effects of maternal age and resource availability on the life courses of the offspring were further studied in the evolutionary background condition, in which offspring had equal chance to experience one of the five types of resource environment at each time step (i.e., the variable environment). Survival was lower for offspring born from mothers from low resource environments (Fig 2A). The proportion of variation in offspring survival (from total variation in a Cox proportional hazard test) explained by maternal resource availability in the evolutionary background condition was 1.2%, and the power to detect a statistically significant difference between offspring from mothers with resource level of 0.1 and offspring from mothers with resource levels 0.3, 0.5, 0.7, and 0.9 was 15%, 48%, 79% and 81% respectively (based on bootstrap of a sample size per treatment of 200 individuals). The maternal age effect on the rate of ageing of offspring was also evident under the evolutionary background condition, but this effect was very small for mothers with biological ages between 0 and 1200 and became apparent only when mothers had reached a high biological age (> 1600; Fig 2B). In this situation, the proportion of the total variation in offspring survival explained by maternal age was 1% and the power to find a decreased survival in offspring of mothers with higher biological ages, relative to a biological age of 0, was 3%, 6%, 7% and 66% for maternal ages of 400, 800, 1200 and 1600 respectively.

We found that the direct environmental effect of constant resource availability on the rate of ageing was large compared to the effect of the resource environment of the mother (Fig 2C). In these simulations, offspring experienced one of five patch types constantly through the rest of development, and the survival curves are therefore clustered into five distinct groups. Within these five classes, only the offspring born from very old mothers had a higher rate of ageing (Fig 2D). When the effect sizes of maternal environment and maternal age were compared to the effect of offspring environment, roughly 60% of the total variation was explained by the resource environment of the offspring, while only 0.6% was explained by maternal environment and maternal age in the separate simulations. The power to detect significant differences between treatment groups of offspring with resource availability was in all cases 100%, while for maternal effects of nutrient environments it was 5%, 16%, 42% and 43% for maternal environments of 0.3, 0.5, 0.7 and 0.9 (relative to maternal environment 0.1) and 4.6%, 5.4%, 8.4% and 45.2% for maternal ages 400, 800, 1200 and 1600 (relative to maternal age 0), respectively.

The results shown here when individuals dispersed at every time-step (thus having the opportunity to experience a patch of different food quality, i.e. the background evolutionary conditions) were qualitatively similar to those when offspring dispersed only once, either at birth or as adults after reaching the age of maturity (S1 and S2 and S3 Figs). The results were consistent both for mortality rates comparable to the results presented in Fig 1 and Fig 2 and for different values for resource availability in addition to those chosen here (Fig F in S1 File). However, in environments with dramatically higher mortality rates age- and resource-specific allocation patterns change considerably, which affects the extent and direction of maternal effects on offspring ageing (S1 and S2 and S3 Figs). The age-specific allocation pattern varies with ageing parameter *κ* (Eqs [1] & [3]; Fig G in S1 File), in a similar way as effects of age-independent mortality rate (compare to S1 Fig). Similar results were obtained when *κ* was varied for post-natal stages only (Eq [1], Fig H in S1 File) or for adult only (Eq [3], Fig I in S1 File). Changing the autocorrelation of the environmental quality did not change the results much (Fig J in S1 File), while developmental time has a large effect on the relative allocation to reproduction (Fig K in S1 File). With shorter developmental time, the relative fitness of juveniles increased (compared to an adult) which led to increased allocation to reproduction and a lack of difference of relative allocation between environments. However, this model was not specifically designed to study this question and we leave further development and interpretation of these results to future work.

## Discussion

Maternal effects on offspring ageing have been reported in both invertebrates and vertebrates, including humans [25,28,46,51,67,68]. Here we show that such effects can be produced by a simple extension of a disposable soma model of ageing. The important insight is that maternal investment into offspring can affect the biological age of offspring at birth (which indicates higher levels of accumulated damage) and hence their age-dependent mortality. Maternal effects on offspring ageing therefore evolve as a consequence of maternal age and maternal environmental effects on reproductive investment. The magnitude of maternal effects depends on how the biological age at birth affects environment- and age-specific resource allocation. Our model derived optimal maternal investment in offspring across maternal age and environmental contexts. Low maternal resource availability and high maternal age both generally reduced investment in offspring and hence created maternal effects on lifespan. However, we also found an interaction between maternal resource availability and age. The effect of maternal resource availability on offspring lifespan was more pronounced in young mothers. Furthermore, the effect of maternal age was most pronounced in high resource environments. Overall, the strength of maternal effects was low compared to direct environmental effects.

Motivated by empirical data, we were specifically interested in the effect of maternal resource availability and maternal age on offspring ageing. Our model shows that variation in reproductive investment across levels of resource availability and maternal lifespan are likely to have small, but detectable, effects on offspring biological age at birth and hence their age-dependent mortality and lifespan [43,54]. This is consistent with long-term studies of individually marked animals that have established maternal effects on offspring ageing. For example, in great tits, male offspring raised in nests where environmental quality (and hence parental food provisioning) is high had increased lifespan compared to males raised under low quality conditions [69]. The natal environment did not affect the lifespan of females in this study, but in the same population daughters born from older mothers aged faster [25]. Offspring in red-billed choughs had a shorter lifespan when born from older parents [14]. Furthermore, this negative correlation was strongest in environment where offspring had low survival chances [14]. Recently, in wild sparrows it was found that offspring of older parents produced less recruits, which resulted in lower lifetime reproductive success [28]. These results are in line with the predictions from our model and show that maternal effects on ageing are not simply a consequence of variation in offspring size, but perhaps reflect incurred somatic damage via variation in maternal investment in maintenance (e.g. [42,70]). Our model could be extended to incorporate sex differences in reproductive allocation in order to explore why these parental age effects are often sex-specific (e.g. [28,71]).

Our model also predicts that such effects will be non-linear when very old females are included. This will be difficult to detect in field studies, however, because in natural populations very old individuals are rare [72]. One possible example is described in free-living Soay sheep where parasite egg count in lamb faeces increased more steeply within the oldest maternal age categories ([73] see also the study of great tits [25]). A similar drastic shift at high maternal age has been found in experiments on the very short-lived soil mite *Sancassania berlesei*. Fitness did not differ for offspring born from 2 and 5 days old mothers, but there was a severe decrease in fitness for offspring born from 5 and 8 days old mothers [74]. As a result, selective breeding from old mothers should result in progressively reduced lifespan across generations as originally described and discussed by Lansing [26].

Our prediction that both maternal age and maternal resource availability should affect ageing of offspring is consistent with data from putative markers of biological age. A number of recent studies on birds and mammals, including humans, have shown that poor or stressful conditions early in life can have strong effects on telomere length, which is associated with lifespan [42,44,75]. Similarly, variation in maternal phenotype or environment has been shown to affect the degree of oxidative damage of offspring (see [46]), which is believed to be a direct cause of ageing [31,41,76,77]. If such effects are a direct outcome of optimal variation in maternal investment into reproduction, as in our model, it follows that there may be an interaction between maternal resource availability and maternal age on offspring lifespan. Although our model is highly idealized, this prediction appears quite robust to parameter settings and could therefore be subject to empirical test. One possible example of such an interaction was recently described in the butterfly *Pieris brassicae*. Stressed females produced offspring with equal survival regardless of maternal age, while in the non-challenged group offspring from older mothers had lower survival [78]. While our model focused on nutritional stress, it could be extended to other types of stressors, under the assumption that mothers can ‘shield’ offspring from damage by increasing investment [37]. Our approach could thus be used to generate predictions for how, for example, telomere shortening and other biomarkers should be affected by maternal stress, infection and age in wild animals [48,68].

Although maternal effects on ageing are expected to evolve under the disposable soma theory, we show here that they are small (in terms of explained variation) compared to direct environmental effects. This is expected as the time for which offspring depend on maternal investment for somatic maintenance is typically short compared to their total lifespan. We believe that a small effect size is consistent with empirical data across studies. For example, the effect of nutrition later in life had a larger effect on mortality compared to nutrition during early life (including *in utero* conditions) in a preindustrial human population [79], which is comparable to our results. However, firm conclusions will have to await further explicit tests of the relative contribution of maternal and direct environmental effects on lifespan (e.g. [69]). Furthermore, the results in our model may be somewhat biased towards small effects. This is because we assumed that a unit of investment into maintenance has the same positive effect at each age (but see Supporting Information for evidence that this only has minor effects on allocation to offspring maintenance). In reality, the rapid growth and differentiation early in life may be associated with particularly severe risk of damage. Since in mammals, this period coincides with that of maternal dependence, the cost of reduced maternal investment may therefore contribute disproportionally to ageing. This generates the empirical prediction that, in species with higher average maternal investment, reduction in maternal quality (due to poor resources or senescence) would incur a stronger effect on offspring ageing (estimated as life span or using biomarkers), than a similar reduction in maternal quality in species with reduced overall maternal investment.

The basic results and the model’s ability to generate testable predictions suggest to us that further expansion of this model framework or alternative models of ageing (e.g., antagonistic pleiotropy) applied to maternal effects will be worthwhile. As usual for theoretical models, we made a number of simplifying assumptions here that do not apply to specific biological scenarios. These include the decision to consider a fixed number of time steps until maturation and that there is no further cost of differential growth in terms of damage. Both of these assumptions could be relaxed or modified in future work. Of particular interest is to include an opportunity for costly compensatory growth, which has been shown to affect lifespan in previous models using the disposable soma framework [34,35]. Since compensatory growth incurs an additional cost of a poor start in life, we suggest that it may amplify maternal environmental effects on lifespan. Our model is also specific in that it considers how maternal effects evolve in a fine-grained resource environment where individuals encounter many different levels of resource availability (we also evaluated the consequences of this for ‘experiments’ that are conducted in a single environment and environmental autocorrelation within an individual’s lifetime; see SI). One interesting aspect of a less fine-grained environment is that the environment experienced by offspring may be predictable based on the environment experienced by mothers [58]. If this is the case, maternal effects may enable adaptive plasticity. A model by Mangel [36] has explored the consequences of adaptive plasticity for damage accumulation and ageing under the disposable soma framework. The results suggest that mismatch accelerates ageing and this may be applicable also to adaptive plasticity mediated by maternal effects.

The driver of maternal effects in our model is reproductive investment. Thus, there will be a range of situations that can modify the relationship between maternal environment, maternal phenotype (including age), and offspring biological age. For example, mothers may face a trade-off between the number and quality of offspring, which under the assumptions of this model could affect offspring lifespan both within and between reproductive bouts [80, 81]. Relaxing assumptions about the number of offspring produced at any given time point will also enable generation of predictions for how maternal effects should influence reproductive ageing in terms of reduced fecundity (e.g. [82]).

In conclusion, we have shown that the disposable soma theory of ageing in principle can explain maternal age and maternal resource effects on offspring lifespan. Our model predicts that offspring from mothers with low resource availability will have shorter lifespan. We also predict that maternal age will affect offspring longevity, such that older mothers generally produce offspring that age faster. The strength of the effects is expected to vary across life histories and environments, but is likely to be quite small relative to direct environmental effects (at least in the general conditions that relate to our model set-up). Predictions appear to be broadly consistent with empirical data, and we suggest that tailoring the life history of the model to systems that are studied empirically would be a useful way to put the disposable soma theory for maternal effects on ageing to the test.

## Supporting Information

S1 Data All data from project. (7Z)

S1 Fig Figure of relative allocation dependent on age dependent and age independent mortality, dispersal for all individuals. (PDF)

S2 Fig Figure of relative allocation dependent on age dependent and age independent mortality, dispersal newborn offspring. (PDF)

S3 Fig Figure of relative allocation dependent on age dependent and age independent mortality, dispersal adults. (PDF)

S1 File Supplementary information for van den Heuvel, English & Uller 'Disposable soma theory and the evolution of maternal effects on ageing'. (DOCX)
