# Supplementary figures and images for: Disposable Soma Theory and the Evolution of Maternal Effects on Ageing
Source: PLoS One. 2016 Jan 11;11(1):e0145544. doi: 10.1371/journal.pone.0145544 (PMC4709080; doi:10.1371/journal.pone.0145544)

# Dispersion all individuals

## Age dependent mortality

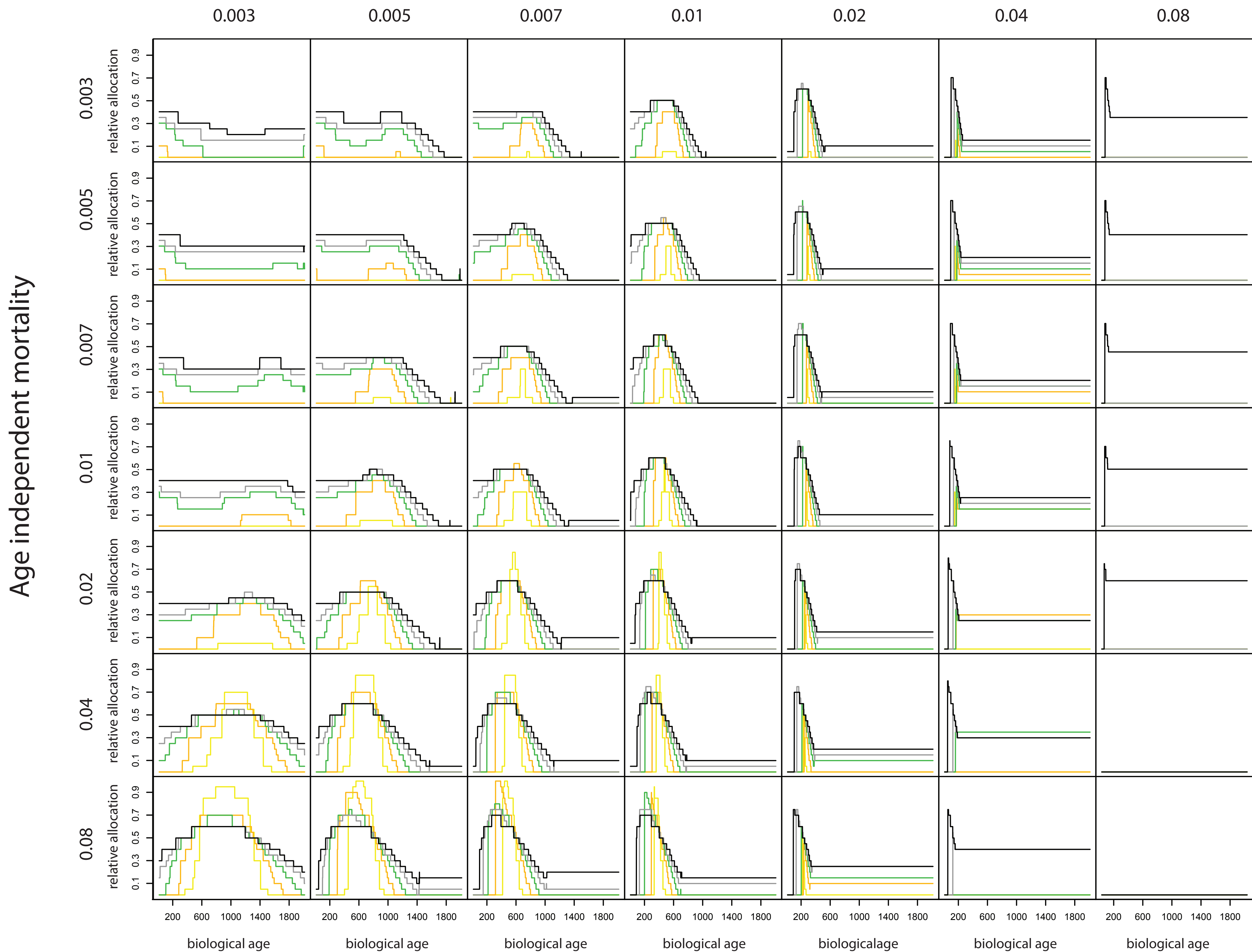

Supplement: S1 Fig — (PDF) [file pone.0145544.s002.pdf]

Dispersion newborn juveniles

Age dependent mortality

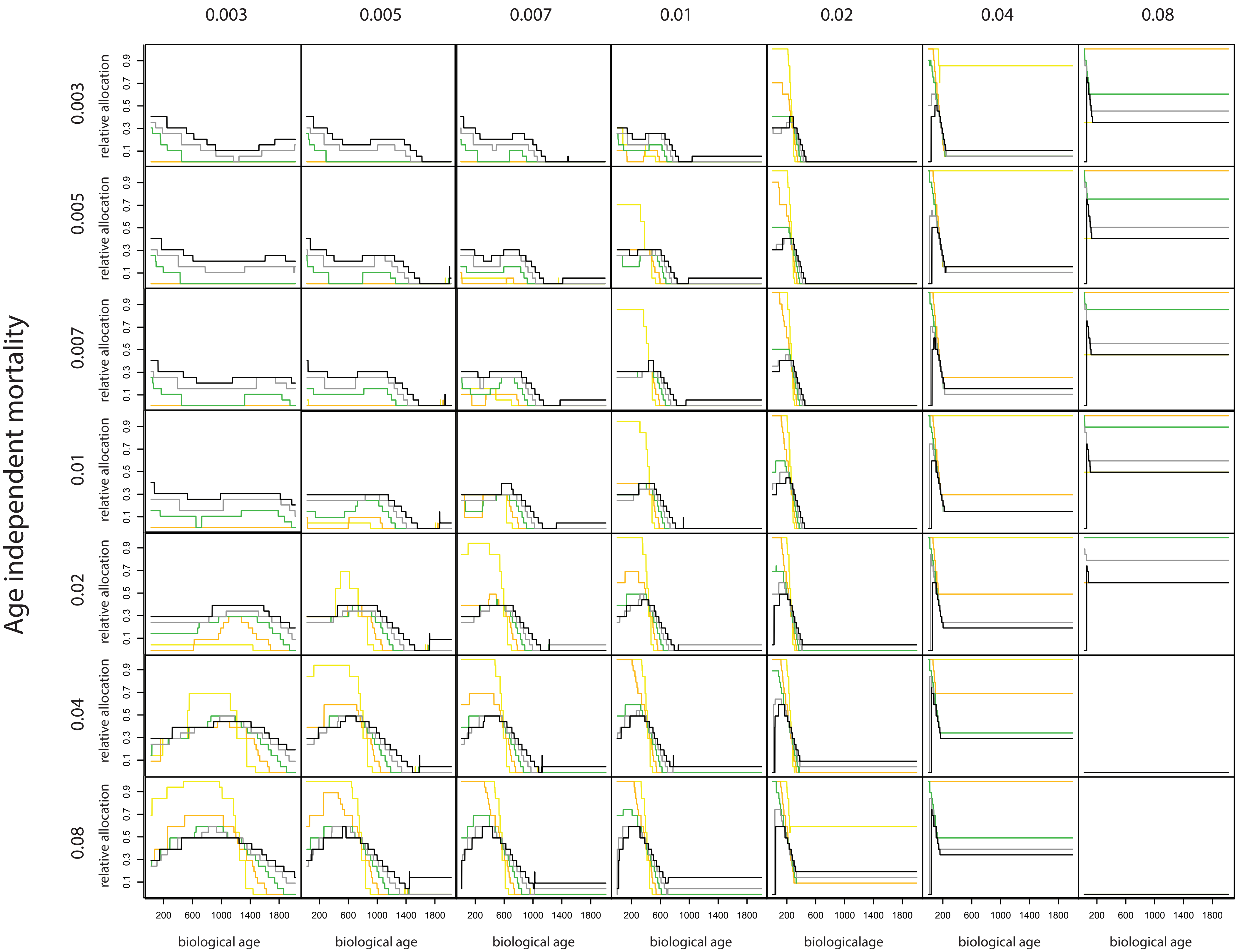

Supplement: S2 Fig — (PDF) [file pone.0145544.s003.pdf]

# Dispersion adults

## Age dependent mortality

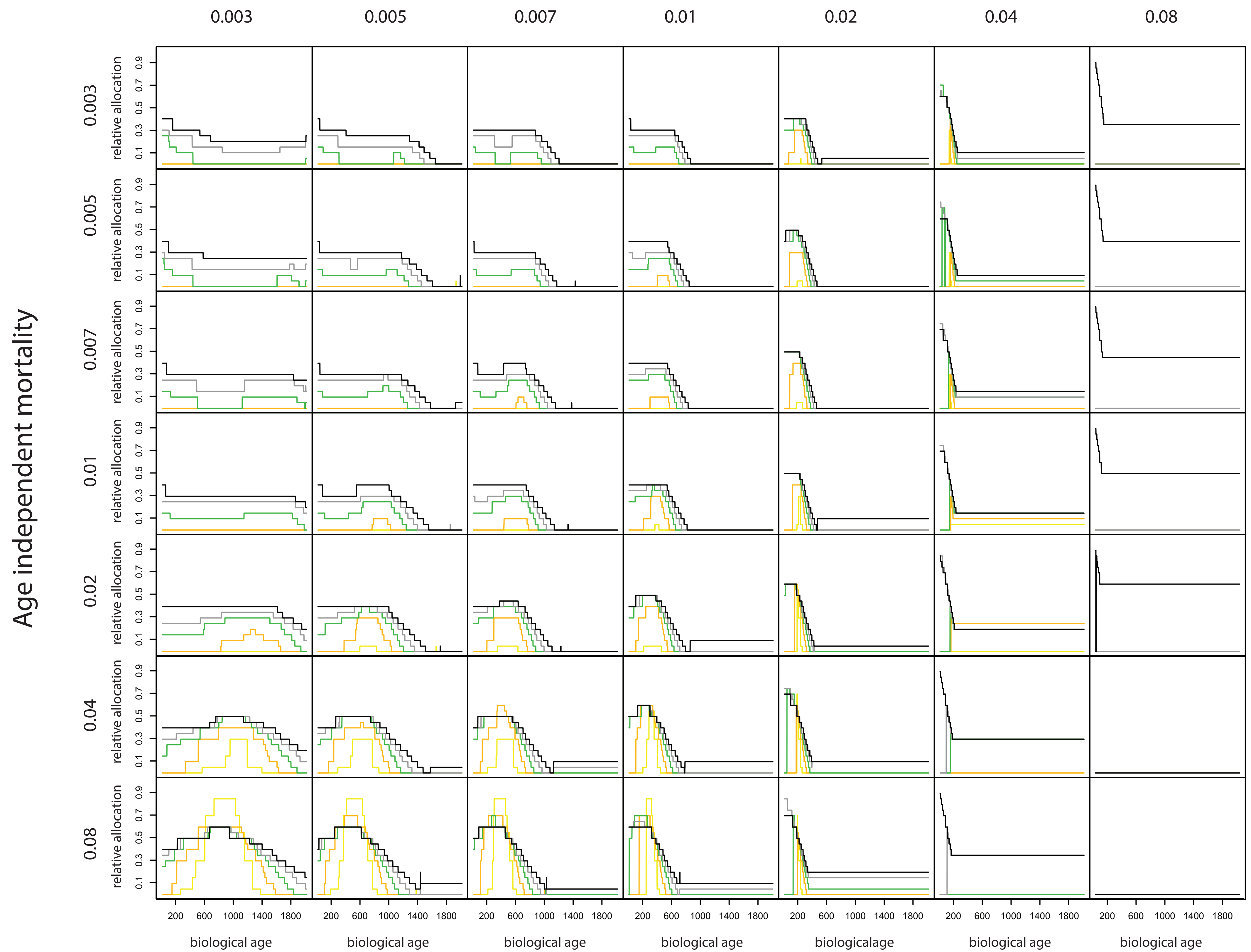

Supplement: S3 Fig — (PDF) [file pone.0145544.s004.pdf]
