# Supplementary material for: Disposable Soma Theory and the Evolution of Maternal Effects on Ageing
Source: PLoS One. 2016 Jan 11;11(1):e0145544. doi: 10.1371/journal.pone.0145544 (PMC4709080; doi:10.1371/journal.pone.0145544)
Supplement: S1 File — (DOCX) [file pone.0145544.s005.docx]

**Supplementary information for van den Heuvel, English & Uller 'Disposable soma theory and the evolution of maternal effects on ageing'**

**Supplement 1: The calculation of fitness**

We consider an optimal decision based on the state of the organism (*D*) and the environment which varies in resource availability. A trade-off is introduced between maintenance (and repair) and reproduction. Fitness is not simply a function of the number of offspring produced but complicated by the fact that more allocation of resources leads to a higher quality offspring with lower damage accumulated. We consider *V(D,E)* to be the reproductive value of an individual with damage *D* in patch type *E* of which there are five different values that refer to the acquisition of resources within the patch. When an individual allocates less resource to offspring, their offspring have lower survival throughout life since they have a higher damage state. At each time step, we consider the choice to allocate a proportion *q* into maintenance and (1-q) into reproduction. For an adult, reproductive value for an individual with damage *D* at patch *E* is

V(D,E) = (exp-M)*(V(D’,E’)+V(D_0_,E’)) (1)

where D’ is the damage level of the adult in the next time step and D_0­_ is the damage state of the offspring. In general, we can state that V(D’,E’) decreases independently with higher damage and with lower quality patches. Similarly, V(D_0_’,E’) decreases with higher damage and lower patch qualities. Let us assume therefore that V(D,E) during adulthood can be written as,

$V_{A}\left( D,E \right)=c_{A}D^{d_{A}}E^{e_{A}}$ (2)

Where *c*, *d*, and *e* are constants and *c* and *e* have values higher than 0 and *d* lower. The subscript A refers to ‘Adult’. The reproductive value of juveniles is different, since they do not yet reproduce. Therefore, we denote *V_J_(D,E)* as the reproductive value of juveniles with subscript J:

$V_{J}\left( D,E \right)=c_{J}D^{d_{J}}E^{e_{J}}$ (3)

Furthermore mortality *M* depends on damage and two constants *m_1_* and *m_2_* which can be considered as damage-independent and damage-dependent mortality parameters. Therefore mortality is

$M=m_{1}D^{m_{2}}$ (4)

In total every time step an adult acquires *P* resources of which a proportion *q* is allocated to maintenance and (1-q) to reproduction. When more is allocated to maintenance, the damage of the adult increases less, while when more is allocated to reproduction, damage of the offspring increases less. In both cases we consider the increase of damage as,

${\Delta D}_{t}(U)=\kappa\frac{exp(\omega U)}{\beta+exp(\omega U)}$ (5)

where *U* is the absolute amount of resource allocated to maintenance or reproduction. Therefore adult damage increase is denoted as ΔD_t_(qP) while for offspring this is ΔD_t_((1-q)P). Substituting these equations into equation (1) produces the fitness function,

$V\left( D,E \right)=exp(-\left( m_{1}\left[ D+{\Delta D}_{t}\left( \mathrm{qP} \right)]^{m_{2}} \right) \right)(c_{A}[D+{\Delta D}_{t}\left( \mathrm{qP} \right)]^{d_{A}}E^{e_{A}}+c_{J}[{\Delta D}_{t}\left( (1-q)P \right)]^{d_{J}}E^{e_{J}})$ (6)

Next, we determine the shape of this function, for values of *q* between 0 and 1 (i.e., the proportion of resources that can be allocated).

There are three possible outcomes under the current assumptions. This is easiest visualized by realizing that we sum up an increasing function (with more allocation to maintenance, reproductive value of the adult part increases) with a decreasing function (with more allocation to maintenance the juvenile reproductive value decreases). These functions increase and decrease monotonically. The first outcome is that total reproductive value is a monotonically decreasing function of *q*, and fitness has an optimum somewhere between 0 and 1 for values of *q* or fitness is an increasing function of *q*. In any case, the fitness landscape is smooth, and there is a single optimal value which can be readily obtained by dynamic programming. Figure 1 shows the outcome of calculating the values of eq. (6) over different values of *q* between 0 and 1 for five different cases of *d_J_.* In every of the five cases there is a clear optimum. This optimum differs across resource environments, E, because the effect of damage on reproductive value of the juvenile (*d_J_*) is different. These fitness landscapes change when comparing young and old mothers (compare figure A to figure B), but each has a single optimum. In figure C the effect of resource environment is simulated in a similar way.

**Figure A. Fitness landscapes for young mothers.** Examples of fitness landscapes for young mothers (D=10, 1% of maximum) as a function of the allocation parameter *q* (x-axis) and five different levels of the effect of juvenile reproductive values. If reproductive value of juveniles is strongly positively affected by resource allocation (indicated by dJ>>) it is more advantageous to allocate more to reproduction, while the opposite is true when the effect of allocation has a more modest effect on juvenile reproductive value.

**Figure B. Fitness landscapes for old mothers.** Examples of fitness landscapes for old mothers (D=1800, 90% of maximum) as a function of the allocation parameter *q* (x-axis) and five different levels of the effect of juvenile reproductive values. If reproductive value of juveniles is strongly positively affected by resource allocation (indicated by dJ>>) it is more advantageous to allocate more to reproduction, while the opposite is true when the effect of allocation has a more modest effect on juvenile reproductive value. Note differences between Figure S1.1 and Figure S1.2.

**Figure C. Fitness landscapes for mothers of different resource levels.** Relative allocation to maintenance (x-axis, left low, right high) for young mothers (D=10. 1% of maximum) in different resource environments (E). Fitness is relative to the highest fitness within each resource environment (because absolute fitness depends on the environmental resource availability). At low resource availability, fitness is maximized at the highest level of maternal maintenance and therefore minimum (baseline) allocation to reproduction. As resource availability increases, the relationship transforms into a parabolic shape.

**Supplement 2: Verification of optimal strategies using simulations**

We simulated populations according to the optimal strategy derived using dynamic programming. We calculated the rate of increase for this strategy 100 times for 100 time steps and calculated the population size average over these 100 simulations. We subsequently ‘mutated’ this optimal strategy by randomly increasing or decreasing age-specific allocation by steps of 0.05 and repeating the calculation of optimal population size. We performed this analysis by mutating the strategies 29 times, in total, starting from the optimal strategy we performed 30 simulations. This was repeated 14 times. Figure D shows the results.

The optimal strategy has a rate of increase parameter of 1.048 (R_0_ per time step). This is close to the optimal strategy found by the dynamic programming algorithm and suggests that dynamic programming reasonably approximates a global maximum. This conclusion is also supported from comparing the allocation strategies from dynamic programming to the simulation results (Figure E).

­­

**Figure D. Boxplots of simulation performed using genetic simulation.** At simulation 1, simulations are initiated using the optimal allocation level obtained by dynamic programming. This optimal allocation strategy is followed by individuals for 100 time steps and the rate of increase estimated from the population size. This was repeated 100 times. A new simulation is started by mutating the optimal allocation pattern for the different environments and biological ages.

**Figure E. Comparison of genetic simulation and dynamic optimization results.** Optimal allocation to reproduction for mothers from the genetic simulation with the highest rate of increase (left) compared to the results obtained by dynamic programming with different biological ages (x-axis) and patch qualities.

**Supplement 3: Additional results for different parameter settings**

Mortality rates and dispersing stages

Below, we present results of our model when we vary our assumptions of age-dependent and age-independent mortality depending on whether dispersal occurs throughout life, once at birth, or during the adult phase only.

**Figure S1. Effect of age-independent and age-dependent mortality (dispersal across life).** Maternal allocation patterns across different mortality settings depending on maternal biological age (x-axis panels) and maternal resource availability (differently colored lines, in yellow, E=0.1, orange, E=0.3, green, E=0.5, grey, E=0.7, black, E=0.9). Allocation is expressed on the y-axis as relative allocation to reproduction (1-q, see eq. [2b]). Rows represent the effect of variation in age-independent mortality (basal mortality, µ_b_). Columns represent the effect of variation in age-dependent mortality (mortality dependent on damage i.e. D, referred to as biological age, ,_d_). The results for mortality levels 0.005 for both i_b_ and h_d_ are described in the main text. These panels indicate the results for the optimal allocation patterns for organisms that randomly move between environments at each time point (i.e., between every reproductive bout) with a probability of 0.2 to encounter any of the five different nutritional environments.

Conclusion: The effect of maternal age on reproductive allocation is strong and increases with age-dependent mortality. There is an interaction between maternal resource environment and maternal age on the relative allocation to offspring maintenance, which increases in strength in environments with higher age-independent mortality. Because the effect of offspring biological age at independence also depends on the absolute amount of resources allocated, offspring from low nutrition mothers consistently have lower age-dependent survival and hence shorter lifespan.

**Figure S2. Effect of age-independent and age-dependent mortality (dispersal at birth).** Maternal allocation patterns are shown across different parameter settings for maternal biological age (x-axis panels) and maternal resource availability (differently colored lines, in yellow, E=0.1, orange, E=0.3, green, E=0.5, grey, E=0.7, black, E=0.9), when dispersal is limited to the time of birth. Allocation is expressed on the y-axis as relative allocation to reproduction, i.e., allocation to offspring maintenance (parameter 1-q, see eq. [2b]). Rows represent the effect of variation in age-independent mortality (basal mortality, p_b_). Columns represent the effect of variation in age-dependent mortality (mortality dependent on damage i.e. D, referred to as biological age, e_d_). The results for mortality levels 0.005 for both e_b_ and h_d_ are described in the main text.

Conclusion: When dispersal occurs only at birth (i.e., a single resource environment is encountered at each time point following dispersal, see S2 Fig), the relative maternal allocation remains the same as when dispersal occurs at every time step for low age-independent mortality. Under high age-independent mortality, there is a reversal of allocation pattern such that young mothers allocate relatively more to offspring quality in the low resource environment, while old mothers allocate relatively more to offspring quality in the highest quality patches. Thus, when maternal survival is high, but the resource environment of the offspring is uncertain, females increase allocation to offspring at the cost of their own maintenance in low quality environments.

**Figure S3. Effect of age-independent and age-dependent mortality (dispersal as adult).** Maternal allocation patterns are given across different parameter settings for maternal biological age (x-axis panels) and maternal resource availability (differently colored lines, in yellow, E=0.1, orange, E=0.3, green, E=0.5, grey, E=0.7, black, E=0.9), with adult dispersal at each time step but no juvenile dispersal. Allocation is expressed on the y-axis as relative allocation to reproduction, i.e., allocation to offspring maintenance (parameter 1-q, see eq. [2b]). Rows represent the effect of variation in age-independent mortality (basal mortality, p_b_). Columns represent the effect of variation in age-dependent mortality (mortality dependent on damage i.e. D, referred to as biological age, µ_d_). The results for mortality levels 0.005 for both e_b_ and h_d_ are described in the main text.

Conclusion: The results for adult-only dispersal are similar to those when both adults and juveniles disperse (see S3 Fig). Consequently, maternal investment in offspring, and hence offspring biological age at birth, vary more across different juvenile dispersal assumptions than for adult dispersal assumptions.

Acquisition rates

We then consider the effect of varying additional model parameters and assumptions, including the rate of acquisition of resources, the rate of increase in ageing with damage, the extent of environmental autocorrelation between time-steps and the length of the juvenile period.

**Figure F. Effect of variation in acquisition rates.** Optimal allocation to reproduction for mothers with different biological ages (x-axis) and patch qualities with (left) less variation of acquisition compared to the main paper results and (right), an overall increase of acquisition rate. On the left patch quality variation is reduced (yellow = very low [E=0.3], orange = low [E=0.4], green = average [E=0.5], grey = high [E=0.6] and black = very high [E=0.7]) and on the right quality of all patches has been increased (yellow = very low [E=0.8], orange = low [E=0.9], green = average [E=1.0], grey = high [E=1.1] and black = very high [E=1.2]). In the main paper the value for E in the first patches are 0.1, 0.3, 0.5, 0.7 and 0.9.

Conclusion: Reduced variation between environments in resource availability reduces variation in relative allocation to reproduction, particularly among young mothers (Figure F).

Maximum increase of ageing

**Figure G. Effect of rate of increase in ageing per time step, κ (for mothers and offspring).** The values of *κ* are indicated by the titles of the panel graphs. Optimal allocation to reproduction for mothers with different biological ages (x-axis) and patch qualities (yellow = very low [E=0.1], orange = low [E=0.3], green = average [E=0.5], grey = high [E=0.7] and black = very high [E=0.9]). Different panels indicate runs where parameter *κ* was altered, the value indicated by the panel titles. In the main paper, *κ* is kept at 1000.

Conclusion: Increased *κ* led to decreased allocation to reproduction in early life. In the main paper data was discussed for when parameter *κ* was set at 1000. In this case, allocation at low biological age is relatively stable but decreases late in life (after a biological age of around 1000). Furthermore, allocation to reproduction is increased at high resource availability. Here, higher *κ* affects the age- and environment-specific allocation, such that allocation to reproduction gradually increases to a maximum at intermediate biological age, after which it decreases until death. Similar patterns are found when *κ* is varied only for the maternal effect on biological age at birth (Figure H) or for the post-natal period (Figure I).

**Figure H. Effect of rate of increase in ageing per time step, κ (for maternal effect on biological age at birth).** Optimal allocation to reproduction for mothers with different biological ages (x-axis) and patch qualities (yellow = very low [E=0.1], orange = low [E=0.3], green = average [E=0.5], grey = high [E=0.7] and black = very high [E=0.9]). Different panels indicate runs where parameter *κ* was altered, but only for biological age at birth, the value indicated by the panel titles. In the main paper, *κ* is kept at 1000.

**Figure I. Effect of rate of increase in ageing per time step, κ (for post-natal period).**. Optimal allocation to reproduction for mothers with different biological ages (x-axis) and patch qualities (yellow = very low [E=0.1], orange = low [E=0.3], green = average [E=0.5], grey = high [E=0.7] and black = very high [E=0.9]). Different panels indicate runs where parameter *κ* was altered, but only for the post natal stages, the value indicated by the panel titles. In the main paper, *κ* is kept at 1000.

Environmental autocorrelation

**Figure J. Effect of environmental autocorrelation.** Optimal allocation to reproduction for mothers with different biological ages (x-axis) and patch qualities (yellow = very low [E=0.1], orange = low [E=0.3], green = average [E=0.5], grey = high [E=0.7] and black = very high [E=0.9]). In the main paper, the environment changes between time steps. However, it is also possible to make it more likely that consecutive time steps have the same environment (which could arise because of spatial structure, for example). Different panels indicate runs spatial correlation varies between zero and one as indicated by the titles of the panels. When spatial correlation is zero, each environment is equally likely at each time step (i.e., the probability of having the same environment is 0.2).

Conclusion: Environmental autocorrelation affects the age-specific relative allocation, such that mothers of intermediate age invest the most in reproduction in low resource environments particularly as the environment becomes more autocorrelated.

Developmental time

**Figure K. Effect of developmental time.** Optimal allocation to reproduction for mothers with different biological ages (x-axis) and environment (yellow = very low [E=0.1], orange = low [E=0.3], green = average [E=0.5], grey = high [E=0.7] and black = very high [E=0.9]). Different panels indicate runs in which developmental time (number of time steps before a juvenile becomes an adult) varies from 20 (as in the main paper) to 4, as indicated by the titles of the panels.

Conclusion: As developmental time decreases, the reproductive value of juveniles, compared to adults, increases. Hence, it becomes more advantageous generally to invest in reproduction (Figure K). This also results in loss of environment-specific allocation strategies, probably because there no longer are any differences in reproductive value of offspring from different resource environments.
